# Potential of Climate Change and Herbivory to Affect the Release and Atmospheric Reactions of BVOCs from Boreal and Subarctic Forests

**DOI:** 10.3390/molecules26082283

**Published:** 2021-04-15

**Authors:** H. Yu, J. K. Holopainen, M. Kivimäenpää, A. Virtanen, J. D. Blande

**Affiliations:** 1 Department of Environmental and Biological Sciences, University of Eastern Finland, P.O. Box 1627, 70211 Kuopio, Finland; hao.yu@uef.fi (H.Y.); jarmo.holopainen@uef.fi (J.K.H.); minna.kivimaenpaa@uef.fi (M.K.); 2 Department of Applied Physics, University of Eastern Finland, P.O. Box 1627, 70211 Kuopio, Finland; annele.virtanen@uef.fi

**Keywords:** drought, herbivory, secondary organic aerosols, volatile organic compounds, warming, boreal forest, subarctic

## Abstract

Compared to most other forest ecosystems, circumpolar boreal and subarctic forests have few tree species, and are prone to mass outbreaks of herbivorous insects. A short growing season with long days allows rapid plant growth, which will be stimulated by predicted warming of polar areas. Emissions of biogenic volatile organic compounds (BVOC) from soil and vegetation could be substantial on sunny and warm days and biotic stress may accelerate emission rates. In the atmosphere, BVOCs are involved in various gas-phase chemical reactions within and above forest canopies. Importantly, the oxidation of BVOCs leads to secondary organic aerosol (SOA) formation. SOA particles scatter and absorb solar radiation and grow to form cloud condensation nuclei (CCN) and participate in cloud formation. Through BVOC and moisture release and SOA formation and condensation processes, vegetation has the capacity to affect the abiotic environment at the ecosystem scale. Recent BVOC literature indicates that both temperature and herbivory have a major impact on BVOC emissions released by woody species. Boreal conifer forest is the largest terrestrial biome and could be one of the largest sources of biogenic mono- and sesquiterpene emissions due to the capacity of conifer trees to store terpene-rich resins in resin canals above and belowground. Elevated temperature promotes increased diffusion of BVOCs from resin stores. Moreover, insect damage can break resin canals in needles, bark, and xylem and cause distinctive bursts of BVOCs during outbreaks. In the subarctic, mountain birch forests have cyclic outbreaks of Geometrid moths. During outbreaks, trees are often completely defoliated leading to an absence of BVOC-emitting foliage. However, in the years following an outbreak there is extended shoot growth, a greater number of leaves, and greater density of glandular trichomes that store BVOCs. This can lead to a delayed chemical defense response resulting in the highest BVOC emission rates from subarctic forest in the 1–3 years after an insect outbreak. Climate change is expected to increase insect outbreaks at high latitudes due to warmer seasons and arrivals of invasive herbivore species. Increased BVOC emission will affect tropospheric ozone (O_3_) formation and O_3_ induced oxidation of BVOCs. Herbivore-induced BVOC emissions from deciduous and coniferous trees are also likely to increase the formation rate of SOA and further growth of the particles in the atmosphere. Field experiments measuring the BVOC emission rates, SOA formation rate and particle concentrations within and above the herbivore attacked forest stands are still urgently needed.

## 1. Introduction

Climate change effects on natural and man-made ecosystems are dominated by warming, which has resulted in global temperature increases of approximately 0.8 °C since the late 19th century. During the same period, Arctic areas depending on measurement site have warmed by 2 to 3 °C [1]. At northern latitudes, the main drivers of rapid warming are increased ocean temperature that leads to decreased annual sea ice cover and reduced global albedo, and earlier terrestrial snow melt [2,3]. These drivers will affect other drivers of warming, such as increased emissions of the greenhouse gas methane from the oceans [3] and thawing permafrost [4,5,6]. Emissions of biogenic volatile organic compounds (BVOC) are strongly temperature dependent with daily and annual variation of emission rates [7]. Thus, warming in the boreal and subarctic areas will also increase the emission rates of BVOCs from soil and vegetation, peatlands [7,8], and lake waters [7]. In forests, the foliage [9], trunks [10], cones [11], roots, and rhizosphere [12,13] of trees, leaf and needle litter deposited on soil [14] and tree stumps [15] can be sources of BVOCs.

In polar areas and at high altitudes of mountainous areas, a cold temperature is the main factor limiting the diversity of organisms. However, climate warming, e.g., in the subarctic, might reduce species richness when the most cold-tolerant species face higher competition due to the spread of less-cold tolerant species [16]. In mountainous areas, larger plants, such as shrubs and trees, have shifted altitudinally upwards to former grasslands [17]. In northern forests, deciduous forest trees have shifted toward higher latitudes [18,19].

Climate change with warming and consequential changes in precipitation will result in the shift of vegetational zones toward the north. These shifts include the expansion of woody shrubs into tundra ecosystems [20,21], a shift of the conifer boreal zone northwards to subarctic areas [18,22,23], and an increased proportion of deciduous trees within the boreal conifer forest [18,19]. Insects are the major poikilothermic animal group to take advantage of a warming climate. There is already evidence that rapidly reproducing and invasive insect herbivores have become more common at northern latitudes and attack forest trees at higher rates causing serious outbreaks and forest decline in boreal conifer forests [24,25,26] and in subarctic deciduous forest [27,28].

According to a recent bibliometric analysis covering the years 1991 to 2018, a general trend in the BVOC literature has been for a growing focus on the assessment of plant stress responses and the atmospheric chemistry of BVOC emissions [29]. In this review we focus on the latest research on the emissions of non-methane BVOCs from boreal and subarctic forest trees under a changing climate. Our review also covers the effects of a warming climate on herbivore pressure and how this may affect the oxidation reactions of BVOCs in the atmosphere that lead to secondary organic aerosol (SOA) formation. Feedback loops between ecosystem change and atmospheric chemistry are still poorly known. The combination of ecological research and atmospheric chemistry research may provide explanations for BVOC-controlled biosphere-atmosphere interactions and lead to better tools for mitigation of ecological effects of global warming. A good example is from a recent study testing the effects of a defoliating moth outbreak on the atmospheric quality above a mountain birch forest [30]. On the basis of earlier laboratory experiments [31], it was expected that highly reactive herbivore-induced BVOC emissions may result in elevated SOA concentrations in the atmosphere. This was not the case, but the total aerosol particle concentrations were elevated for up to a few years after the infestation, which was expected to indicate delayed defense responses, such as the stimulated regrowth of mountain birch [30].

## 2. Major BVOCs Emitted from Boreal and Subarctic Forest Ecosystems

Boreal and subarctic forests are the most northern forest ecosystems before the arctic tundra. The major BVOCs found in these environments are the same low-molecular-weight and mostly lipophilic volatile molecules that plants and microbes emit in a strongly temperature-dependent way in warmer ecosystems [32,33,34]. However, low- and semi-volatile compounds might differ in their atmospheric and ecological behavior during the short and relatively cold summer. Semi-volatiles such as oxygenated sesquiterpenes become non-volatile and condense on plant surfaces during colder night temperatures before being re-emitted to the atmosphere from both the emitter plant and surfaces of neighbouring plants as temperatures warm the following day [35,36].

The major chemical groups of BVOCs emitted by plants include terpenoids comprising the C5 isoprene and C10 monoterpenes synthesised by the 2-C-methyl-D-erythritol 4-phosphate (MEP) pathway in the plastids and C15 sesquiterpenes formed via the mevalonate pathway (MVA) in the cytosol, green leaf volatiles (GLV) formed via the oxylipin–lipoxygenase (LOX) pathway and aromatic compounds (benzenoids and phenylpropanoids) formed via the shikimate (SHI) pathway [32,33,37]. These compounds are effectively sampled with adsorbent-filled cartridges and analysed by gas chromatography-mass spectrometry (GC-MS) in the laboratory [38]. Several studies have shown that the constitutive emission rates of these compounds from the foliage [9,37,39,40], bark [41,42], and root systems [12,13,43] of trees are related to abiotic conditions. Many of these compounds are also emitted at an order of magnitude higher rates from herbivore or fungal pathogen-damaged plants [44,45,46,47,48,49]. Some of the compounds, such as GLVs and sesquiterpenes (SQT), are mostly detected in emissions after feeding damage [50]. High emissions of monoterpenes (MT) are typical of conifer forests and are released from stored and exposed resin. After insect feeding or mechanical damage, the resin flow from damaged needle stumps can release MTs at vastly higher rates than from intact twigs [10].

**Figure 1:**
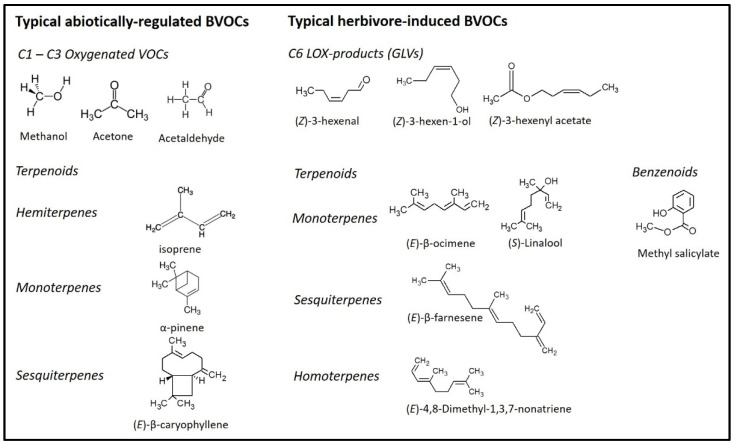
Examples of molecular structures of some major BVOCs emitted by boreal and subarctic forests trees. Constitutive BVOC emission rates are often regulated in response to abiotic factors such as temperature and light. Herbivore-induced BVOC emissions have strong response to herbivore feeding damage.

In recent years, studies using proton transfer reaction–mass spectrometry (PTR-MS) have indicated that forest trees can emit significant amounts of smaller, oxygenated volatile organic compounds (OVOC), which comprise non-methane C1–C3 molecules such as methanol, acetaldehyde, and acetone [42,51,52,53,54,55]. These emissions are related to changes in plant physiology and availability of water [42]. Emissions of highly volatile and water soluble OVOCs from stem bark indicate that OVOCs are leaked into the atmosphere when they are transported in the xylem sap from their sources in tree roots to the foliage [42,52]. Mechanical damage to foliage has been shown to significantly increase methanol and acetaldehyde emissions from leaves [51]. Emissions of herbivore-induced BVOCs are the result of a combination of mechanical plant tissue damage, together with insect secretions such as saliva and the enzymes therein [56]. The induction process of these BVOCs is described in more detail in Section 5. Structural examples of constitutively emitted (but abiotically controlled) BVOCs and herbivore-induced BVOC molecules are given in Figure 1.

## 3. Effects of Warming and Light on BVOC Emissions from Trees and Rhizosphere

Temperature is well known to play an important role in BVOC biosynthesis and emissions [57]. A rising temperature, up to approximately 40 °C, can increase the activity of the enzymes involved in BVOC biosynthesis [58]. However, severe heat stress can result in cellular lesions and a reduction in photosynthesis that may not recover upon return to cooler temperatures, thus reducing de novo produced BVOC emissions [59]. Increasing temperatures may also increase the vaporization of BVOCs leaking from storage pools (ducts or glands) [60]. Therefore, temperature and light conditions during BVOC sampling have a substantial effect on calculated emission rates. As a consequence, the investigation of elevated temperature effects on plant emissions is complicated and longer-term climate warming effects should be separated from local weather condition effects during sampling. When the emission rates per unit of plant dry weight or leaf area are presented, they can be standardized to a certain target temperature, most commonly 30 °C [57]. However, standardization steps should be carefully considered as they may not be suitable to colder regions that seldom reach temperatures as high as 30 °C [61].

BVOC emissions are predicted to increase in response to long-term warming. To detect the effect of warming, plant BVOC emissions have been collected in experiments with plants grown in plots with either ambient temperatures or regulated warming with temperatures maintained at a target elevated level using heaters, e.g., modulated infra-red (IR) radiators [62,63]. The effects of warming on coniferous species have been investigated in Scots pine (*Pinus sylvestris*) and Norway spruce (*Picea abies*). In *P. sylvestris*, heating has been shown to have mixed results; an increase of 1 °C maintained with IR-heaters in an open-field has been shown to cause two-fold to four-fold increases in emissions of non-oxygenated monoterpenes (MT-no), oxygenated monoterpenes (MT-ox), and SQTs by seedlings [9]. However, in a study by Tiiva et al. [64] a greater warming increase (2 °C above the ambient level) showed unclear effects on BVOC emissions. In *P. abies*, warming (1.3 °C above the ambient level) has been shown to increase emissions of MT-ox [65].

The effects of warming on BVOC emissions have also been studied in broad-leaved species. In European aspen (*Populus tremula*) saplings, an increase of 1 °C significantly increased emissions of total monoterpenes (MT) and green leaf volatiles (GLV) [62]. In a latter study, isoprene (IP) emission rate was shown to be increased by warming of 2 °C above the ambient level [66]. Studies on birch saplings (*Betula pubescens* var. *pumila*) also identified increased IP emissions in response to warming of 3–4 °C [67]. In a study of *Betula pendula* Roth an increase in emissions of mono- and homoterpenes, SQTs and compounds other than terpenes (GLVs and methyl salicylate (MeSA)) was observed with warming of 0.8–1 °C [68].

Warming affects BVOC emissions in different ways for different leaf types. In deciduous leaves without specific BVOC storage structures, emissions are affected by warming due to temperature-dependent biosynthesis. By comparison, BVOC emissions of conifer needles are affected by a combination of temperature-dependent biosynthesis and vaporization. Rhizosphere BVOC emissions can potentially be affected by warming via two main mechanisms; first they can be directly affected by environmental effects on the soil and roots, and secondly they can be indirectly affected, whereby emissions belowground are influenced by effects on the aboveground parts of the plant. The effects of warming on rhizosphere BVOC emissions have been investigated in a coniferous and a broadleaved boreal forest tree species. An elevated temperature of 1 °C above the ambient level had only weak effects on *P. sylvestris* seedlings [13,43]. Moreover, warming had no effect on the emissions of *P. tremula* [66].

In the boreal forest zone, warming can alter the availability of water and nutrients and prolong the growing season [69], which is expected to alter the tree species composition. In Finland, the annual mean temperature in 2100 is predicted to be 4 °C higher than in 1990 [18]. By the same time, the countrywide proportions of *P. sylvestris* and *P. abies* are predicted to be reduced from 49 to 12% and from 38 to 33%, respectively, with increased dominance of the birches *B. pendula* and *Betula pubescens* from 13 to 55% [18]. The predicted changes in tree species composition, have been calculated to increase MT and IP emissions from 950 to 1108 kg km^−2^ y^−1^ and from 132 to 214 kg km^−2^ y^−1^, respectively [18]. However, predictions did not consider potential acclimation of tree species to warming, differences in responses to warming by different species (Table 1), or effects of increased herbivory under climate warming (see Section 4, Section 5 and Section 6).

Examples of tree species distributions moving northwards from boreal forests toward the subarctic and arctic tundra include the larch species of North America [70] and Siberia [71]. Siberian larch (*Larix sibirica*) and Dahurican larch (*Larix dahurica*) are dominant tree species in the large Siberian boreal forest ecoregion known as the Taiga [71,72]. BVOC emissions of these two larch species consist of approximately 90% monoterpenes, with the remainder including sesquiterpenes that could account for up to 10% of the total emission in summer time [72]. The American larch (*Larix laricina*) is a tree line species in the boreal forest of Canada [70] and also has a high monoterpene content [73]. Thus, in future, larch species have the potential to become significant monoterpene and sesquiterpene emitters in the current arctic and subarctic tundra area.

## 4. Effects of Climate Warming on Density and Distribution of Herbivores

Many tree-damaging forest insects have a cycle of several years between reaching their outbreak population densities (pest status) within their natural geographical ranges. This cyclicity is linked to variation in the top-down control (by natural enemies) and bottom-up control (by resource limitation and host plant quality) [74,75]. Annual variation in weather conditions will affect these controlling factors, but the weather also directly influences the physiology and propagation rate of herbivorous insects [76]. Increases in mean temperature because of climate warming affect the warming-induced distribution shift of forest insects towards high latitudes [24,26] and high altitudes [76]. Thus, in the future, a higher risk of insect outbreaks in boreal and subarctic forests will come from more frequent outbreaks of native species and additional outbreaks by invasive exotic forest insects.

Some of the major outbreak species of boreal and subarctic forests have become more common because of climate warming (Table 2). Some species are already native outbreak species that only occasionally reach outbreak levels in the cooler climate, for example the bark beetle *Ips typographus* [77] and the sawfly *Neodiprion sertifer* [78]. Other species such as *Lymantria monacha* and *Lymantria dispar* are invasive, e.g., in the boreal zone of Finland, and their population densities may reach outbreak levels in the future [24].

*I. typographus* attacks are predicted to increase on *P. abies* [26], with increased effective temperature sums coinciding with more favorable conditions for swarming. Areas with favorable temperature sums for the complete development of bivoltine populations of *I. typographus* (>1500 degree days (DD)) shifted northwards by an average of 450 km during the entire study period of 1960–2016 [26]. Massive 2.5 M ha outbreaks of the Siberian silk moth *Dendrolimus sibiricus* have shifted northward by up to 500 km in the Siberian boreal conifer forest during the last 40 years with the shift promoted by droughts and an increase in the temperature sum [81,82]. Higher winter temperatures and decreased summer precipitation are the main drivers of the range expansion of Mountain pine beetle, *Dendroctonus ponderosae*, towards the north in the USA and Canada [84,85]. Major defoliator species that exhibit a warming-related distribution shift towards the north in European Boreal forest include the Nun moth (*L. monacha*) and the Gypsy moth (*L. dispar*) [24]. Higher winter survival of eggs and improved dispersal and reproduction success of adult moths are the main drivers for the northward distribution of *L. monacha* [25]. The critical temperature for survival of *L. monacha* eggs in tree bark cracks is −29.5 °C. On the southwestern coast of Finland, temperatures have not dropped below this lethal limit since the year 1987 [25].

*N. sertifer* overwinters as eggs deposited in pine needles. Winter temperatures below −35 °C limit egg survival leading to larger larval populations and an increase in outbreak potential after mild winters [78]. The great web-spinning pine-sawfly *Acantholyda posticalis* overwinters as a larva in soil and in recent years more frequent outbreaks have occurred in pine forests of milder coastal areas of the Baltic sea [83].

## 5. Herbivore Feeding and Oviposition Effects on BVOC Emissions

The percentage of the boreal forest area affected by biotic stress annually was calculated to average 4% between 1988 and 2013 [86]. Insect herbivory contributed most to the total observed damage [86]. Feeding or oviposition by herbivores leads to mechanical damage of plant cells including structural degradation of membranes. Chewing and sucking feeding modes, oviposition, and oral and ovipositor secretions elicit a signal transduction cascade that activates transcription factors of the WRKY gene family [87,88,89]. These transcription factors control phytohormones (salicylic acid, jasmonic acid, and ethylene) that elicit the production of herbivore-induced BVOCs [87]. In terpene storing species such as conifers, herbivore feeding damage promotes release of constitutively emitted BVOCs, such as monoterpenes that have a several fold increase in emission rate after damage by leaf chewing herbivores [9,10,90]. Herbivore damage to plants has also been shown to increase emissions of GLVs and methyl salicylate (MeSA). The effects of herbivore-feeding on BVOC emissions can vary dramatically depending on the mode of feeding and the part of the plant that is fed upon. In the following paragraphs we detail some of the key plant responses to herbivore-feeding and the effects of herbivores with different feeding strategies (Table 3).

Bark-feeding herbivores are among the most damaging outbreak pests of boreal forests. The large pine weevil (*Hylobius abietis*) is a pest of conifers such as *P. sylvestris* [109]. Several studies have shown that *H. abietis* damage on the stem bark of *P. sylvestris* can cause an increase in both localized and systemic emissions of BVOC, including MTs, SQTs, and GLVs that can increase several-fold after feeding [44,91,92,93,94]. The increase in localized BVOC emissions is mainly due to passive evaporation of compounds from resin accumulated at stem feeding sites [97]. The increased systemic BVOC emissions could have several different explanations, including: (1) BVOC synthesis elicited by a signal transmitted via vascular tissue; (2) BVOC synthesis elicited by a signal transmitted via airborne signals from the damage-site to the foliage; and (3) leakage of BVOCs from branch bark where the wounding increases resin flow from foliage via resin ducts [44]. In addition, BVOCs emitted from damage sites may be adsorbed to and then re-emitted from needle surfaces. *H. abietis* feeding has also been shown to increase BVOC emissions have in *P. abies* [91,95]. However, the most severe pest of *P. abies* throughout Europe is *Ips typographus*, an aggressive beetle species. In a study by Ghimire et al. [96], *I. typographus* attack increased MT emissions from the bark surface by 20-fold. In western North America, the white pine weevil (*Pissodes strobi*) is the most destructive insect pest of Sitka spruce (*Picea sitchensis*) [110]. Feeding by *P. strobi* on bark induces a rapid release of MTs and SQTs from *P. sitchensis* [97]. Bark-feeding herbivores can also induce BVOC emissions indirectly, e.g., the mountain pine beetle (*Dendroctonus ponderosae*) associated fungus *Grosmannia clavigera* substantially increased MT emissions in *Pinus contorta* and *Pinus banksiana* [98]. Bark damaging herbivores also feed on broad-leaved species. *H. abietis*, for example, has been reported to feed on silver birch (*Betula pendula*) bark, but did not affect the BVOC emissions of shoots [50].

Defoliating insects are also major outbreak pests in the boreal and subarctic region. Defoliating pests of conifers, such as the sawflies *Neodiprion sertifer* [10,91] and *Acantholyda posticalis* [9,40], have been shown to induce shoot BVOC emissions during needle-feeding. The needle-feeding weevil, *Strophosoma melanogrammum*, was also found to enhance shoot MT emissions from *P. abies* [99]. The increased BVOC emissions from herbivore-damaged conifer shoots may have resulted from emission bursts caused by the breakage of needle resin canals during the herbivore attack. Defoliating insects can also cause substantial damage to broadleaved plants. Feeding by the autumnal moth, *Epirrita autumnata*, has been shown to increase shoot BVOC emissions from mountain birch [31,100,101], silver birch [102], and hybrid aspen [103,104]. Shoot BVOC emissions were also found to increase in response to feeding damage by *Erranis defoliaria* in silver birch [50] and *Cabera pusaria* in alder (*Alnus glutinosa*) [105]. Herbivore-induced BVOC emissions might be used as a defense against herbivory. For example, there is evidence that aspen produce volatile chemicals upon herbivore damage that attract predators and parasitoids [111,112,113].

It is notable that while not constituting as important pests as outbreaking bark beetles and defoliating insects, piercing-sucking herbivores also infest trees in the boreal and subarctic regions, and have effects on BVOC emissions. Although there have been numerous studies on plants in general [114], very few studies have explicitly investigated the effects of piercing–sucking herbivores on BVOC emissions of boreal trees. Kivimäenpää et al. [106] showed that *P. sylvestris* infested by the aphid *Cinara pinea* Mordvilko, had significantly increased emissions of methyl salicylate (MeSA) and SQTs, but MT emissions were not affected. In a study of *B. pendula* and *A. glutinosa* by Blande et al. [107], some induced terpene emissions were recorded, but the most notable aphid-induced emission was MeSA. These two studies indicate that aphid-feeding characteristically induces emissions of MeSA which appear to be dependent on aphid density [106] and duration of infestation [107].

While the BVOC emissions of aboveground plant parts have typically received greater attention than those emitted belowground, we found four studies addressing the effects of herbivore-feeding on BVOC emissions from the rhizosphere [10,13,43,50]. Leaf feeding by the geometrid moth species *E. defoliaria* Clerck and *Agriopis aurantiaria* Hübner and bark feeding by *H. abietis* had no effect on rhizosphere BVOC emissions from *B. pendula* [50]. Moreover, needle [13] and bark [43] feeding on *P. sylvestris* had no clear effects on BVOC emissions from the rhizosphere. Assessment of herbivore-feeding directly on roots appears to be lacking from the current literature.

In addition to herbivore feeding, oviposition by herbivores can induce BVOC emissions in boreal forest tree species [108]. Oviposition on *P. sylvestris* by the sawfly *Diprion pini* was shown to increase production of MTs [108]. Plant responses to oviposition can directly affect the eggs. Emission of benzyl benzoate, for example, can have an active ovicidal function [115]. Oviposition-induced BVOC emissions can also structure defenses against neonate larvae; for instance, oviposition by the moth species *Chilo partellus* has been shown to attract the larval parasitoid *Cotesia sesamiae* [116]. Likewise, oviposition by the large cabbage white butterfly (*Pieris brassicae*) on the wild crucifer *Brassica nigra* attracted the larval parasitoid *Cotesia glomerata* [117]. In *P. sylvestris*, BVOC emissions induced by *D. pini* oviposition attract the egg parasitoid *Chrysonotomyia ruforum* [108]. However, more studies are needed to investigate BVOC emission responses to oviposition by herbivores in boreal trees.

In general, BVOC emissions from boreal trees were increased by herbivore stress. However, in a natural environment, herbivore stress does not occur in isolation. Trees are exposed to multiple concurrent biotic and abiotic stressors. To date there are few studies on how herbivore-induced BVOC emissions in boreal and subarctic forests are affected by simultaneous abiotic stresses, including potential modifications in a warming climate.

## 6. Combined Warming and Herbivore-Feeding Effects on BVOC Emissions

In the previous section, several examples of herbivore-feeding effects on BVOC emissions were presented. The effects of insect herbivore feeding on BVOC emissions depend on various internal plant and environmental factors [118]. Temperature is the most important abiotic driver of BVOC emissions; it affects the chemical defense strategies of plants and thus influences the capacity of plants to emit BVOCs. An analysis of 18 terpene-storing *Pinus* spp. from altitudinal and latitudinal gradients indicated that species originating from colder high altitudes rely more on constitutive defenses (terpenes produced in storage structures), while species originating from warmer environments rely more on induced defenses (terpenes synthesized after induction by tissue damage) [119]. This may suggest that in warming climatic conditions conifers adapted to cold conditions could become more active producers of induced BVOCs under herbivore attack. Over a longer time frame, the result of a warming climate could be that conifer species and provenances that have stronger induced defense response will prevail in boreal and subarctic forests.

The biotic stress and damage caused by herbivores is strongly influenced by the feeding habits and mode of the herbivore such as chewing, sucking, mining, or gall forming strategies. Host plant responses to these specific damage types is influenced by abiotic conditions during damage, which together influence the dominant BVOC composition in the specific global vegetational zone [120]. For example, isoprene is a major BVOC released by vegetation at latitudes closest to the equator while at higher latitudes monoterpenes are the more dominant BVOC emissions [120,121]. An analysis of the effects of gall-forming insect feeding on plant emissions in low and high latitude vegetation zones indicated a general trend for feeding damage to reduce isoprene emission rates with the relative reduction most distinctive at low latitudes. Monoterpene emissions responded positively to gall-forming insect feeding with the relative change at the same level as for isoprene, but in the opposite direction. The highest increase and relative change were detected for sesquiterpene emissions in tropical areas, while GLVs were emitted by all infected leaves in all regions [120].

The effects of a combination of warming and herbivore pressure on BVOC emissions are most naturally studied in experimental field exposure systems where plants are grown in plots with either ambient conditions or a warming treatment that elevates temperature to a target level. In warming plots, infrared radiators are used to continually expose the canopy to an elevated target temperature for 24 h per day. Limitations in these systems are that most of the studies are conducted using small tree seedlings or saplings. Another approach is to build a closed top [122] or open-top chamber over a single tree and control the temperature inside the chamber and compare chamber-enclosed trees at various temperatures. Investigations of herbivory effects on tree BVOC emissions under these conditions are difficult and there is a risk of unintentional insect outbreaks by typical greenhouse pest insects such as aphids. Closed chamber experiments are more easily regulated for temperature and confounding factors, but are less natural.

In an open-field exposure system with Scots pine seedlings, modest feeding damage (10% of a shoot) by larvae of the sawfly *A. posticalis* in combination with warming (+1 °C addition) did not affect BVOC emissions of Scots pine during the growing season, although both factors separately increased emissions [9]. Substantially elevated monoterpene emissions from warming treatments, where needles developed larger resin canals, were partly masking potential interactive effects [9]. However, in the same study, warming and herbivory from the previous growing season increased emissions of the monoterpene camphene at the beginning of the next growing season, highlighting the potential long-term effects of herbivory under climate warming. Warming can also cause thermal stress to herbivores [123] and a 1 °C warming lasting three growing seasons was shown to reduce feeding by *A. posticalis* and herbivory-induced total MT emissions by 77% [40]. A closed chamber experiment by Tiiva et al. [64] showed that a +2 °C temperature increase and short-term bark herbivory by *H. abietis* increased total MT emissions from foliage of Scots pine seedling growing in N-fertilized (30 kg ha^−1^ year^−1^) soil.

An open field experiment using needle-feeding *A. posticalis* larvae reduced emissions of minor MTs from the rhizosphere where roots were the main emission source, while warming (1 °C in air and 0.7 °C in soil) did not influence the emission response [13]. Another open field experiment examining responses to *H. abietis* weevils showed reductions in emissions of total sesquiterpenes and total non-terpenoid volatiles from the roots in response to herbivory, while warming (0.5 °C in air and 4 °C in soil) reduced emissions of monoterpenes, sesquiterpenes, and total non-terpenoid volatiles [124]. The reductions were not observed when warming and herbivory were combined. In a closed chamber experiment, a short feeding period by *H. abietis* weevils on the bark at the base of the stem reduced some root BVOC emissions, but this reduction was not evident in a +2 °C warming treatment [43]. Thus, herbivory effects seem to generally reduce root BVOC emissions under ambient conditions, but not under warmer climatic conditions.

## 7. Atmospheric Reactions and the Effects of Climate Change

Ecosystem resilience to changes in abiotic conditions is based on the capacity of vegetation to adapt to these changes. BVOCs produced in plant cells are central to plant adaption to temperature and drought stress [60]. BVOC emissions are also part of the biosphere–atmosphere loop where ecosystem BVOC emissions link aerosols and clouds in forest ecosystems [125,126,127,128]. If climate change continues at its current rate, there is a clear risk that resilience of forest ecosystems will be jeopardised resulting in substantial changes to ecosystem structure and function [129].

Common BVOCs belonging to the same chemical group may have high variability in their reactivity with major atmospheric oxidants. Highly reactive compounds degrade the fastest in atmospheric reactions and have short atmospheric lifetimes (Table 4). The molecular structure of each compound affects its reactivity, and the higher the number of double bonds the higher the reactivity with atmospheric oxidants [130,131]. BVOCs released into the atmosphere such as monoterpenes are transformed into low-volatility organic compounds in atmospheric reactions within a few hours [132,133,134,135]. Further oxidation of these first-generation low-volatility compounds produces progressively less-volatile products that will initiate biogenic secondary organic aerosol (BSOA) particle formation and growth by condensation in the atmosphere [132,133,134,135].

As discussed in earlier sections, BVOC emission rates and emission profiles will change in a warming climate due to both increasing temperature and expected increases in herbivore outbreaks. In general, the increase in emission rates will increase the gas phase concentration of BVOC, and hence enhance BSOA formation through atmospheric oxidation processes [138,139]. However, the exact effect of increased BVOC emissions on the formed BSOA mass is complicated and depends strongly on how the BVOC emission profiles will change with increasing temperature in different environments. These complications are due to the reactivities of different BVOCs with atmospheric oxidants and the vapor pressure of the oxidation products formed. Furthermore, the BSOA mass yields depend strongly on the structure and chemical characteristics of precursor BVOCs.

It has been thought, for example, that the sesquiterpene to monoterpene ratio could control the volatility of oxidation products and consequently the BSOA yields, whereby an increasing fraction of sesquiterpenes would decrease the volatility of oxidation products and increase the yield. However, more detailed investigations have shown that, as well as this ratio, the specific type of the terpene whose fraction is changing defines how the volatility of oxidation products and the subsequent BSOA yield changes. Faiola et al. [92] reported increases in SOA yields with an increasing contribution of sesquiterpenes in experiments whereby β-carophyllene contributed most to the sesquiterpene load. However, rather contrasting results by Faiola et al. [140] and Ylisirniö et al. [30] show that when farnesene was the dominant sesquiterpene species, there was a trend for a decrease in SOA yields in photo-oxidation (with both O_3_ and OH present) experiments with an increasing sesquiterpene ratio. The reason for the different behavior of the two sesquiterpene types is based on their molecular structure. Bicyclic compounds, such as β-carophyllene, have one endo- and one exocyclic C=C bond, while acyclic compounds (e.g., farnesene) have four C=C bonds. In the (photo-)oxidation process, the carbon backbone of acyclic compounds is fragmented into smaller pieces because both O_3_ and OH break the C=C bonds efficiently. Just a single oxidation step can decrease the number of carbons from 15 to 5–12 [136,141]. In the case of the bicyclic compounds, fragmentation is expected to be much less prominent [142,143]. Furthermore, it has been shown that changing monoterpene emission profiles due to pine weevil feeding greatly affects SOA mass yields [140]. This observation is supported by results showing that an increased fraction of monoterpenes with endocyclic C=C bonds increases the SOA yield [30]. Measurements of SOA yields of α-phellandrene, which has an endocyclic C=C bond, showed that the SOA yield was about twice that of α-pinene, reaching up to 100% [144].

According to Faiola et al. [92] specific monoterpene emission profiles can play a critical role in controlling SOA yields, particularly when the sesquiterpene to monoterpene ratio is small (less than 0.1). The complexity of oxidation chemistry and yields of different terpene classes and differences between and also within the same terpene classes should be considered in more detail in future studies. In many climate models, α-pinene (monoterpene) and isoprene are considered as model compounds for predicting BSOA formation from plant emissions.

In addition to affecting the overall formation of BSOA, changing BVOC profiles may have an effect on the physical and chemical characteristics of the BSOA formed. It has been shown that precursor VOCs greatly affect, e.g., the hygroscopicity and the physical phase state of SOA particles (e.g., [145,146,147,148]). SOA from sesquiterpene oxidation generally has lower hygroscopicity and maintains a semi-solid or solid physical phase state at higher humidity conditions than monoterpene- or isoprene-derived SOA. Hence the changes in BVOC emissions profiles due to increased temperature and herbivore outbreaks may also affect the cloud condensation nuclei (CCN) and ice nuclei (IN) activity of BSOA. Therefore, changing BVOC emissions might influence atmospheric CCN concentration and consequently the indirect effects of aerosols in two different ways:(1) by changing the amount of BSOA in the atmosphere and (2) by changing the CCN and IN properties of formed SOA.

The observational data for the loadings of BSOA and CCN in the atmosphere demonstrate that increased temperature will likely increase BVOC emissions, the formed BSOA, and hence the CCN concentrations in the atmosphere [138,139,149]. Furthermore, Yli-Juuti et al. [149] have recently shown that in the boreal region temperature driven increases in BSOA loadings result in significant increases in both the aerosol optical depth (AOD) and cloud albedo analyzed using satellite observations.

## 8. Herbivore Damage Effects on Secondary Aerosol Formation in the Atmosphere

Our knowledge of the effects of herbivore-induced emissions of forest trees on atmospheric SOA formation is still sparse [114,135] and has only been experimentally tested in a few studies [30,31,91,92,127,140,150]. Some outbreak species, such as *L. dispar*, consume foliage at a relatively fast rate, which leads to the loss of VOC-emitting leaf material, but also leads to physiological disturbances in intact leaves that reduce photosynthesis. In isoprene-emitting trees, such as *Quercus robur*, isoprene emission is reduced while damaged leaves emit higher rates of herbivore-induced VOCs [151]. Herbivore-feeding on deciduous birch foliage results in emission of highly reactive GLVs within minutes of damage [50], and these emissions are known to be related to SOA formation [152]. *Betula* species are important monoterpene and sesquiterpene emitters also [31,68,153]. Within 24 h of feeding initiation, increased emission of monoterpenes, sesquiterpenes and the homoterpene (*E*)-4,8-Dimethyl-1,3,7-nonatriene (DMNT) are detected [31]. Numerous compounds in the chemical groups are highly reactive with atmospheric oxidants such as ozone [31,154,155].

In a laboratory exposure experiment, deciduous mountain birch BVOC emissions and subsequent SOA particle formation rates and mass yields from reactions with ozone were measured during folivory by an outbreaking geometrid moth *E. autumnata*. The results showed that increased larval density strongly stimulated herbivore-induced BVOC emissions. When compared to controls without damage, a distinctive formation of new particles via nucleation in the reaction chamber was observed, and the SOA mass loadings from damaged seedlings increased by up to 150-fold [31]. However, a later study [30] testing the effects of an *E. autumnata* outbreak on atmospheric quality and aerosol particle concentrations above an attacked mountain birch forest did not find a direct correlation between the larval density and aerosol particle growth rate or the total particle concentration. Interestingly, the analysis of total aerosol particle concentrations suggested that they were elevated for 2–3 years after an initial infestation by the larvae. The authors suggested that it may be an indication of delayed defense responses, such as herbivory stimulated regrowth of mountain birch. However, the damage intensity might have been too low to affect SOA formation to draw any clear conclusions [30]. Among the herbivore induced BVOCs of mountain birch, sesquiterpenes have been shown to have delayed induction, which can occur for a couple of years after the end of an outbreak [156]. In several studies on conifers, biotic stress-induced BVOC emissions have been linked to SOA formation in O_3_ and OH-induced atmospheric reactions [91,114,157]. Feeding by chewing insects induces monoterpene-dominated BVOC emissions at the feeding sites on the plant [10,44] and in distal needles [44]. Emissions of some herbivore-induced sesquiterpenes such as (*E*)-β-farnesene, were only detected from individual needles infested by the needle-feeding spider mite *Oligonychus ununguis*. [158]. Damage by chewing insects will result in resin flow from damaged needle residues, such as needle stumps [10]. Bark feeding insects such as weevils [44,93] and bark beetles [10,159,160] produce significant damage to resin-storing tissues and cause monoterpene-releasing resin flow. Conifer bark-feeding aphids (*Cinara* spp.) induce emission of bicyclic monoterpenes [161], sesquiterpenes [140], and methyl salicylate [106,157].

Temperature is affecting forest BVOC emissions and formation of SOA in the atmosphere and thus warmer and longer summer temperature and extended growing season will increase biogenic SOA formation in the atmosphere [162]. Any specific studies testing the interactions between temperature and herbivore induced BVOC emission rates and SOA formation in boreal forest atmospheres have not yet been published. With agricultural plants, an elevating temperature is positively affecting herbivore-induced BVOC emission rates with the highest in the temperature range of 22 to 27 °C under sufficient daylight [163]. Above 20 °C degree “nightless night” conditions are encountered in the subarctic and northern boreal zones for close to 24 h per day in midsummer.

Joutsensaari et al. [91] analyzed pine sawfly feeding effects on Scots pine needle BVOC emission rates in field experiments and demonstrated 11-fold and 20-fold increases in monoterpene and sesquiterpene emissions, respectively. Laboratory measurements of BVOCs emitted by the bark of Scots pine and Norway spruce seedlings showed 10–50 fold increases in BVOC emissions in response to bark weevil feeding; subsequent reactions with elevated ozone led to 200–1000-fold increases in SOA masses formed compared to SOA masses from reactions with BVOCs emitted by undamaged seedlings. On the basis of these results, a global-scale model projecting a 10-fold increase in monoterpene emission in 10% of the boreal conifer forest area showed significant increases in aerosol masses and a 45% increase in aerosol-cloud condensation-nuclei (CNN) concentrations [91]. These results were verified with satellite monitoring data of aerosol optical density that have shown an increased density of aerosols in the atmosphere above conifer forests attacked by mountain bark beetles in Canada [91]. SOA formed from BVOC reactions were separated from aerosol particles released, e.g., from wild fires, although in some active wild fire periods separation is not particularly efficient. However, forest fires and their aerosol production are partly an indirect result of trees dying in the older beetle outbreak areas. Most importantly, aerosols formed from burning contain black carbon and, thus, have a warming effect in the atmosphere while SOA originating from BVOC emissions mostly act as a sunscreen and has a cooling effect in the atmosphere [164].

The 10 fold increase in reactive BVOC emissions after bark beetle damage and significant increase in SOA mass in atmospheric reactions leading to increases in CNN concentration in the atmosphere [91], suggests that after extensive insect outbreaks in conifer forests there could be climatic-cooling via secondary aerosol and cloud formation. The rate of isoprene emission has been linked to the suppression of new particle formation by scavenging hydroxyl radicals that would react with other terpenes [48]. In boreal conifer forest, the proportion of isoprene emissions is lower than, e.g., from the subarctic peatlands where isoprene could be the dominant BVOC [7] and emissions are increased by warming [8]. Hence, due to high isoprene emissions from peatland sites the induced BVOC emissions from defoliating moth outbreaks on mountain birches in the subarctic might not result in distinctive SOA and CCN formation [30] as in boreal conifer forest after insect outbreaks.

## 9. Conclusions and Future Directions

There is a clear need to better understand the adaptation of boreal and subarctic forest ecosystems to a warming climate. BVOCs are a crucial part of intra- and inter-species communication in forest ecosystems, as well as a major component of biosphere-atmosphere interactions. This means that dramatic disturbances in ecosystem function under climate change may disturb the biosphere–atmosphere feedbacks and then loosen or reinforce the control of climatic trends, such as warming. A good example of the ecosystem change–climate feedback is the loss of permafrost in the Arctic, Antarctic, and alpine regions under climate warming. The flux of major greenhouse gases CO_2_, CH_4_, and N_2_O from soil to the atmosphere will increase substantially during permafrost thawing [165], which has the potential to directly accelerate climate warming. Permafrost thawing may also increase BVOC emissions from soil in the Arctic, but increased microbial activity in the upper soil layer will consume these non-methane BVOCs and regulate their release to the atmosphere [166].

Herbivore-feeding and oviposition have been reported to affect BVOC emissions. To date, the majority of herbivores studied have consumed aboveground parts of plants and BVOC responses aboveground have been focused upon. There are, as yet, few experiments that have investigated the effects of feeding by herbivores either above or belowground on the emissions from the roots and rhizosphere. *H. abietis*—commonly studied for its effects on aboveground plant parts—has been observed to feed [167] and oviposit [168] in root bark, which is likely to affect BVOC emissions [43]. To gain greater insight into the full effects of herbivores and climate on biosphere–atmosphere feedbacks, greater attention should be paid to belowground processes induced by herbivores.

It is also important to note that few studies have considered potential invasive herbivore species in predicting future BVOC scenarios. Invasion of flying herbivores is much more rapid than invasion of new plant species and invasive herbivores may reach outbreak levels in future conditions. While invasive herbivores may outbreak on plant species currently present in the more northern areas, over a longer time scale new invasive plant species will have their own herbivores arriving. Hence, more studies of how warming and potential invasive herbivore species affect BVOC emissions will provide important information in this topic. Since earlier studies under open field conditions have typically investigated one temperature elevation level, there is a need to gain information on response thresholds by conducting warming experiments with several temperature elevations levels in interaction with herbivory. It is known that after emission BVOCs will influence SOA formation, which will further affect climate, but knowledge of warming- and herbivore-induced BVOC impacts on atmospheric SOA formation is still incomplete.

## Figures and Tables

**Table 1:** Examples of warming impacts on emissions of isoprene (IP), non-oxygenated monoterpenes (MT-no), oxygenated monoterpenes (MT-ox), monoterpenes (MT-no + MT-ox, MTs), sesquiterpenes (SQT), green leaf volatiles (GLV) and Methyl salicylate (MeSA). ‘+’ shows the size of increasing warming effects; ‘ne’ means no warming effects.

Tree Species	Warming Treatment	Responding Compounds	Change in Temp. Standardized Emission Rate	Reference
Shoot				
*Pinus sylvestris*	Ambient +1 °C	MTs	+200~400%	[9]
		SQTs	+200~400%	[9]
*Pinus sylvestris*	Ambient +2 °C	MTs	ne	[64]
		SQTs	Only found in warming	[64]
*Picea abies*	Ambient +1.3 °C	IP	ne	[65]
		MT-ox	PCA result: +	[65]
		MTs	ne	[65]
		SQTs	ne	[65]
*Populus tremula*	Ambient +1 °C	IP	ne	[62]
		MTs	+300%	[62]
		GLVs	+400%	[62]
*Populus tremula*	Ambient +2 °C	IP	+56%	[66]
		MTs	ne	[66]
		SQTs	ne	[66]
		GLVs	ne	[66]
*Betula pendula* Roth	Ambient + 0.8~1 °C	MTs + homoterpenes	+400%	[68]
		SQTs	+400%	[68]
		GLVs + MeSA	+40%	[68]
*Betula pubescens* var. *pumila*	Ambient + 3~4 °C	IP	+70%	[67]
Rhizosphere				
*Pinus sylvestris*	Ambient + 1 °C	MTs	ne	[13]
*Pinus sylvestris*	Ambient +1 °C	MTs	ne	[43]
*Populus tremula*	Ambient +1 °C	IP	ne	[66]

**Table 2:** Some major defoliating and bark/trunk damaging outbreak species of boreal trees and species showing expansion towards the north under climate warming to date. Damage type; D = defoliator, T = trunk and bark damage.

Herbivore Species	Damage Type	Host Tree Species	References
European pine sawfly, *Neodiprion sertifer*, Geoffrey	D	Scots pine, *Pinus sylvestris* L.	[78]
Autumnal moth, *Epirrita autumnata*, Bork.	D	Mountain Birch, *Betula pubescens* var. *pumila*, (N. I. Orlova) Hämet-Ahti	[79]
Winter moth, *Operophtera brumata*, L.	D	Mountain birch, *B. pubescens* var. *pumila*, (N. I. Orlova) Hämet-Ahti	[80]
*Ips typographus*	T	Norway spruce, *Picea abies*, Karst.	[26]
Siberian silkmoth, *Dendrolimus sibiricus* Tschetv.	D	Several conifer species, Abies, *Larix*, *Picea* and *Pinus* spp.	[81,82]
Nun moth, *Lymantria monacha* L.	D	Mainly on conifers	[24]
Gypsy moth (*L. dispar* L.)	D	Mainly on deciduous trees	[24]
Great web-spinning pine-sawfly *Acantholyda posticalis* Mats	D	Scots pine, *P. sylvestris* L.	[83]
Mountain pine beetle, *Dendroctonus ponderosae*, Hopkins	T	Pines, *Pinus* spp.	[84]

**Table 3:** Examples of insect herbivory impacts on emissions of non-oxygenated monoterpenes (MT-no), oxygenated monoterpenes (MT-ox), monoterpenes (MT-no + MT-ox, MTs), sesquiterpenes (SQT), green leaf volatiles (GLV) and Methyl salicylate (MeSA). ‘+’ shows the size of increasing warming effects; ‘ne’ means no warming effects.

Tree Species	Herbivory	Responding Compounds	Change in Temp. Standardized Emission Rate	Reference
Shoot				
Bark Damaging Herbivore-Feeding/Conifer and Broadleaf Tree Systems				
Scots pine *Pinus sylvestris*	Large pine weevil *Hylobius abietis*	MTs	+280~400%	[44]
		SQTs	+290~700%	[44]
Scots pine *Pinus sylvestris*	Large pine weevil *Hylobius abietis*	MTs	+1697%	[91]
		SQTs	+357%	[91]
Scots pine *Pinus sylvestris*	Large pine weevil *Hylobius abietis*	MTs	+4224%	[92]
		GLVs	+114%	[92]
Scots pine *Pinus sylvestris*	Large pine weevil *Hylobius abietis*	MTs	ne	[64]
		GLVs	ne	[64]
Scots pine *Pinus sylvestris*	Large pine weevil *Hylobius abietis*	MTs	+9000%	[93]
Scots pine *Pinus sylvestris*	Large pine weevil *Hylobius abietis*	MTs	+300%	[94]
		SQTs	+800%	[94]
Norway spruce *Picea abies*	Large pine weevil *Hylobius abietis*	MTs	+1078%	[91]
		SQTs	+7300%	[91]
Norway spruce *Picea abies*	Large pine weevil *Hylobius abietis*	MTs	+97~744%	[95]
		GLVs	ne	[95]
		SQTs	+4355~5471%	[95]
Norway spruce *Picea abies*	Spruce bark beetle *Ips typographus*	MTs	+2000%	[96]
		SQTs	ne	[96]
Sitka spruce *Picea sitchensis*	Pine weevils *Pissodes strobi*	MTs	+650%	[97]
		SQTs	+1190%	[97]
Mountain pine beetle (*) *Dendroctonus ponderosae*	Lodgepole pine *Pinus contorta*	MTs	+12,000%	[98]
Mountain pine beetle (*) *Dendroctonus ponderosae*	Jack pine *Pinus banksiana*	MTs	+350%	[98]
Silver birch *Betula pendula* Roth	Large pine weevil *Hylobius abietis*	MTs	ne	[50]
		SQTs	ne	[50]
		GLVs	ne	[50]
Foliage Damaging Herbivore-Feeding/Conifer and Broadleaf Tree Systems				
Scots pine *Pinus sylvestris*	Pine sawfly *Neodiprion sertifer*	MTs	+981%	[91]
		SQTs	+988%	[91]
		GLVs	+487%	[91]
		MeSA	+500%	[91]
Scots pine *Pinus sylvestris*	Pine sawfly *Neodiprion sertifer*	MTs	+1400%	[10]
		SQTs	+700%	[10]
		GLVs	+1300%	[10]
Scots pine *Pinus sylvestris*	Pine sawfly *Acantholyda posticalis*	MT-no	+550~2100%	[40]
		MT-ox	+0~910%	[40]
		SQTs	+560~1100%	[40]
		GLVs	+650~920%	[40]
Scots pine *Pinus sylvestris*	Pine sawfly *Acantholyda posticalis*	MTs	ne	[9]
		SQTs	+300%	[9]
Norway spruce *Picea abies*	Needle-eating weevils *Strophosoma melanogrammum*	MTs	+300~2000%	[99]
Mountain birches *Betula pubescens* var. *pumila*	Autumnal moth *Epirrita autumnata*	MTs	+470%	[100]
		SQTs	+117%	[100]
		GLVs	+195%	[100]
Mountain birch *Betula pubescens* var. *pumila*	Autumnal moth *Epirrita autumnata*	MTs	+1000%	[31]
		SQTs	+200%	[31]
		GLV	+800%	[31]
Mountain birch *Betula pubescens* var. *pumila*	Autumnal and winter moth *Epirrita autumnata* and *Operophtera brumata*	MTs	+200~5200%	[101]
		SQTs	−30~+500%	[101]
		GLV	−70~+3050%	[101]
Silver birch *Betula pendula Roth* *(clones 4 and 80)*	Autumnal moth *Epirrita autumnata*	MTs	+1100%	[102]
		SQTs	+140%	[102]
Hybrid aspen *Populus tremula* L. *× P. tremuloides* Michx.	Autumnal moth *Epirrita autumnata*	MTs	+385%	[103]
		SQT	+505%	[103]
		GLV	+1157%	[103]
Hybrid aspen *Populus tremula* L. *×P. tremuloides* Michx (Clone 55)	Autumnal moth *Epirrita autumnata*	MTs	+%	[104]
		SQTs	+422%	[104]
Hybrid aspen *Populus tremula L. × P. tremuloides Michx* (Clone 100)	Autumnal moth *Epirrita autumnata*	SQTs	+570%	[104]
Silver birch *Betula pendula* Roth	Geometrid moth *Erannis defoliaria* Hübner	GLVs	+900%	[50]
Black alder *Alnus glutinosa*	Geometrid moth *Cabera pusaria*	MTs	+940%	[105]
		SQTs	+200%	[105]
		GLV	+2000%	[105]
Piercing-Sucking Herbivore-Feeding/Conifer and Broadleaf Tree Systems				
Scots pine *Pinus sylvestris*	Large pine aphid *Cinara pinea* Mordvilko	MTs	ne	[106]
		SQTs	+582%	[106]
		MeSA	+7413%	[106]
Silver birch *Betula pendula* (Roth)	Aphid *Euceraphis betulae* (Koch.)	MeSA	+319~1526%	[107]
Black alder *Alnus glutinosa* (L.) (Gaertn.)	Aphid *Pterocallis alni* (DeGeer)	MeSA	+9826%	[107]
Rhizosphere				
Scots pine *Pinus sylvestris*	Pine sawfly *Neodiprion sertifer*	MTs	−80%	[10]
		SQTs	ne	[10]
Scots pine *Pinus sylvestris*	Pine sawfly *Acantholyda posticalis*	MTs	ne	[13]
Scots pine *Pinus sylvestris*	Large pine weevil *Hylobius abietis*	MTs	ne	[43]
Silver birch *Betula pendula* Roth	Large pine weevil *Hylobius abietis*	MTs	ne	[50]
	Geometrid moths *Agriopis aurantiaria* (Clerck) and *Erannis defoliaria* Hübner	MTs	ne	[50]
Oviposition/Conifer Tree Systems				
Scots pine *Pinus sylvestris*	Sawfly *Diprion pini*	MTs	+6%	[108]

(*) = induction by bark beetle associated fungi.

**Table 4:** Examples of calculated atmospheric lifetimes of some constitutively emitted and herbivore-induced BVOCs in reactions with major reactive atmospheric oxidants. BVOC classes: I = isoprene, MT = monoterpene, SQT = sesquiterpene, HT = homoterpene, GLV = C6 green leaf volatile. Major oxidants: OH = hydroxyl radical, O_3_ = ozone, NO_3_ = nitrate radical.

VOCs	BVOC	Lifetimes for Reaction with Oxidants			
	Type	OH ^a^	O_3_^b^	NO_3_^c^	Ref
Constitutively emitted compounds					
Methanol	Oxygenate	12 day	>4.5 year	2.0 year	[130]
Isoprene	I	1.4 h	1.3 day	1.6 h	[130]
3-carene	MT	1.6 h	11 h	7 min	[130]
Limonene	MT	49 min	2.0 h	5 min	[130]
α-Pinene	MT	2.6 h	4.6 h	11 min	[130]
Longifolene	SQT	2.9 h	>33 day	1.6 h	[130]
Typical herbivore-inducible compounds					
*cis*-/*trans*-Ocimene	MT	33 min	44 min	3 min	[130]
β-Phellandrene	MT	50 min	8.4 h	8 min	[130]
β-Caryophyllene	SQT	42 min	2 min	3 min	[130]
β-Farnesene	SQT	1.0 h	14 min		[131,136]
DMNT (4,8-dimethyl-1,3,7 nonatriene)	HT	40 min	60 min	3 min	[137]
*cis*-3-Hexenyl acetate	GLV	1.8 h	7.3 h	4.5 h	[130]
cis-3-Hexen-1-ol	GLV	1.3 h	6.2 h	4.1 h	[130]
cis-3-Hexenal	GLV		2 h		[131]
Methyl salicylate	Aromatics		52 h		[131]

Oxidant concentrations used in calculation [130]: (^a^) Assumed OH radical concentration: 2.0 × 10^6^ molecule cm^−3^, 12-h daytime average. (^b^) Assumed O_3_ concentration: 7 × 10^11^ molecule cm^−3^ (30 ppb), 24-h average. (^c^) Assumed NO_3_ radical concentration: 2.5 × 10^8^ molecule cm^−3^, 12-h nighttime average.

## Data Availability

Not applicable.
